# Development of a pH-Responsive Delivery System Suitable for Naringenin and Other Hydrophobic Flavonoids Using the Interactions Between Basil Seed Gum and Milk Protein Complexes

**DOI:** 10.3390/foods15020201

**Published:** 2026-01-07

**Authors:** Ruwanthi Premathilaka, Matt Golding, Jaspreet Singh, Ali Rashidinejad

**Affiliations:** 1 School of Food Technology and Natural Sciences, Massey University, Private Bag 11222, Palmerston North 4442, New Zealand; 2 Riddet Institute, Massey University, Private Bag 11222, Palmerston North 4442, New Zealand

**Keywords:** basil seed gum, pH-driven flavonoid encapsulation, naringenin, colloidal stability, bioactive delivery, flavonoid–protein interactions

## Abstract

Incorporating hydrophobic flavonoids such as naringenin into food systems is challenging due to their poor water solubility and instability. Effective delivery systems are essential to improve solubility, dispersibility, and controlled release during digestion. This study developed a food-grade encapsulation system using basil seed gum water-soluble extract (BSG-WSE) combined with proteins, sodium caseinate (NaCas) and whey protein isolate (WPI), via pH-driven and mild heat treatments in aqueous media, without the use of organic solvents, to ensure safety and sustainability. BSG-WSE and NaCas were tested at mass ratios of 1:1, 1:3, and 1:5 under pH conditions of 4, 5, and 7, followed by heat treatments at 60 °C or 80 °C for 30 min. The total biopolymer concentrations were 0.15%, 0.3%, and 0.45% (*w*/*v*). The most stable colloidal system was obtained at a 1:1 ratio, pH 4, and 60 °C, which was further evaluated for two additional flavonoids (rutin and quercetin) and with WPI as an alternative protein source. The highest loading capacity (11.18 ± 0.17%) and encapsulation efficiency (72.50 ± 0.85%) were achieved for naringenin under these conditions. Quercetin exhibited superior performance, with a loading capacity of 14.1 ± 3.12% and an encapsulation efficiency of 94.36 ± 5.81%, indicating a stronger affinity for the delivery system. WPI showed lower encapsulation efficiency than NaCas. Ternary systems (BSG-WSE, NaCas, and naringenin) formed under different pH and heat treatments displayed distinct morphologies and interactions. The pH 4 system demonstrated good dispersion and pH-responsive release of naringenin, highlighting its potential as a delivery vehicle for hydrophobic flavonoids. BSG-WSE significantly improved the stability of protein-based complexes formed via pH-driven assembly. Physicochemical characterization, rheological analysis, and release studies suggest that this system is particularly suitable for semi-solid food products such as yogurt or emulsions, supporting its application in functional food development.

## 1. Introduction

Naringenin is well known for its diverse therapeutic effects in cell culture and animal models; however, relatively few clinical studies have been conducted [1,2,3]. This has been attributed to its poor solubility, limited stability, and low bioavailability. Interestingly, its low molecular weight (272.26 g/mol) and lipophilicity facilitate penetration of the blood–brain barrier, based on in situ experiments, thereby exerting neuroprotective effects [4]. Joshi, Kulkarni, and Wairkar [2] discuss how naringenin can impact non-communicable diseases such as cardiovascular diseases, cancer, diabetes, and neurological disorders, as well as its antioxidant potential. Moreover, many studies have reported that naringenin plays an important anti-inflammatory role in COVID-19 [5,6,7].

This bioactive compound, classified as a Class II substance by the biopharmaceutical classification system, is poorly soluble but permeable [8]. In the gut, the unionized form of naringenin predominates, but its low water solubility and lipophilicity lead to liver uptake and conversion into glucuronide intermediates for rapid clearance, reducing its bioavailability [9]. The pKa values of naringenin’s OH groups range between 7–10, requiring a high pH (10–12) for complete dissolution in water [10].

Despite its proven therapeutic benefits, such as antioxidant and anti-inflammatory properties [8], its poor water solubility and stability hinder the food applications and bioaccessibility. Encapsulation can modulate the physicochemical environment, enhancing bioavailability.

Numerous nano-encapsulation and microencapsulation strategies have been reported to enhance the bioavailability of naringenin, including polymeric nanoparticles (synthetic or biodegradable), lipid-based systems (liposomes, phytosomes, nanostructured lipid carriers, emulsions), and inorganic nanoparticles [11]. These techniques include various methods such as pH-driven encapsulation, solvent evaporation, thin-film hydration, spray drying, and combinations of these approaches, like sonication [12,13,14]. Most of these methods rely on organic solvents and synthetic polymers to ensure stability and controlled release. In the food industry, all ingredients must be safe for human consumption and preferably classified as GRAS (Generally Recognized as Safe). Natural ingredients, including proteins and polysaccharides, are essential in meeting safety and regulatory standards. While synthetic polymers offer advantages such as ease of scalability, controlled degradation for sustained release, and compatibility with advanced technologies like electrospinning, they also present notable challenges. These challenges encompass environmental concerns, increased carbon footprints, and potential health risks, such as the formation of toxic degradation products and residual solvents in the body. Conversely, natural polymers are biodegradable, environmentally friendly, and support clean-label formulations, which are increasingly preferred by consumers for their safety and sustainability benefits.

In recent years, there has been a growing interest in protein–polysaccharide–polyphenol ternary systems or complexes as a sustainable means of delivering polyphenolic compounds. Polysaccharides help prevent undesirable aggregation between proteins and polyphenols, while polyphenols, in turn, harness the interactions between proteins and polysaccharides to ensure tighter binding. Polyphenols’ higher affinity for proteins has been extensively studied [13]. Herein, adverse processing conditions should also be minimized (e.g., high temperature). From the product point of view, i.e., flavonoid fortified-functional foods, aspects such as matrix compatibility and high water dispersibility should be ensured. At the same time, the high loading capacity of the system makes the scale-up procedure more economical.

Encapsulation methods, such as the pH-driven method, have been increasingly recognized, and the incorporation of stabilizers such as polysaccharides has been proposed as a strategy to overcome their potential drawbacks, such as sedimentation. A recent study [4] has suggested that ternary systems show superior properties to binary nano delivery systems or individual biomolecules owing to the synergetic effect and altered biological crown states. In contrast, protein binary systems suffer from high susceptibility to biodegradation (enzyme degradation) and limited natural membrane permeability [11,14], and polysaccharide coatings may mitigate such drawbacks.

To the best of our knowledge, the water-soluble component of basil seeds gum or mucilage has not yet been utilized to enhance the stability of protein–polysaccharide–polyphenol ternary complexes. Basil seed gum (BSG) is an edible plant hydrocolloid recognized for its interactions with various protein candidates. It comprises a mixture of water-soluble polysaccharides, water-absorbing polysaccharides, and hydroxyproline-rich proteins. BSG exhibits excellent rheological properties, including emulsification, gelation, and adsorption capabilities, which are comparable to those of commercially available gums [15].

Structurally, BSG is composed of glucomannan, with a glucose to mannose ratio of approximately 10:2, xylan with acid side chains at the C-2 and C-3 positions of xylosyl residues, and a minor fraction of glucan. Research indicates that BSG contains uronic acids, rendering these anionic polysaccharides mucoadhesive [15,16]. The xylan component contributes to the hydrophilic nature of the polysaccharide, allowing it to behave as random coils in dilute solutions [17]. Additionally, BSG can serve as a valuable source of dietary fiber, offering potential health benefits and functional applications in food formulations.

Several studies have indicated BSG’s ability to interact with different protein candidates. For instance, Mortazavi Moghadam et al. [12] used a combination of BSG and casein to synthesize a thin film encapsulating nanogels containing lemon peel oil, demonstrating high mechanical strength and thermal stability. Other studies have analyzed the interactions between BSG and Sodium caseinate (NaCas) in the presence of CaCl_2_ and heat treatment [18], as well as interactions with egg albumin [19], whey protein isolate (WPI) [15,16], and soy protein isolate (SPI) [17] under various heat treatments. These studies highlight BSG’s excellent rheological properties that may be used for the delivery of bioactive compounds.

Although BSG has primarily been utilized in film and fiber formation rather than bioactive encapsulation, most research has involved extracting the entire gum through scraping techniques, followed by filtration and high-speed centrifugation. These processes aim to isolate water-soluble polysaccharides from the crude gum by removing insoluble particles. However, such methods can potentially damage the polysaccharides due to excessive processing, highlighting the need for gentler extraction techniques. As an alternative, a hot water extraction method was employed, which proved to be less aggressive and more cost-effective. This approach offers advantages for large-scale production by providing a more consistent polysaccharide solution that is free of insoluble particles. The resulting uniform charge distribution enhances the suitability for encapsulation procedures, such as pH-driven methods.

In this study, we hypothesized that the water-soluble extract of basil seed gum (BSG-WSE), combined with proteins such as NaCas and WPI, could serve as an effective food-grade delivery system for hydrophobic flavonoids like naringenin. The encapsulation process utilized was a pH-driven self-assembly, primarily relying on electrostatic interactions, hydrophobic forces, and hydrogen bonding. This strategy aims to develop a stable colloidal system capable of delivering naringenin with pH responsiveness and favorable textural properties. It was further hypothesized that such a system could enable targeted delivery in the intestine and colon, following protection in the stomach. The study also examined the effects of pH, biopolymer ratio (BSG-WSE: NaCas), and temperature conditions on the system’s efficacy. Additionally, the research evaluated the potential of this delivery system for other hydrophobic flavonoids, such as rutin and quercetin, and explored the use of WPI as an alternative protein source. This allowed for an assessment of how structural differences among proteins and flavonoids influence the system’s loading capacity and overall performance.

## 2. Material and Methods

### 2.1. Materials

Sweet Italian basil seeds (sourced from Davis Food Ingredients, Palmerston North, New Zealand), sodium caseinate, and whey protein isolates (Fonterra, Auckland, New Zealand) were used in this study. Naringenin, rutin, and quercetin (Sigma-Aldrich, Auckland, New Zealand, purity ≥ 95%) were also utilized. All other chemicals and reagents were of analytical-grade quality, procured from Sigma-Aldrich (Auckland, New Zealand) or Thermo Fisher Scientific (Auckland, New Zealand).

### 2.2. Methods

#### 2.2.1. Extraction of BSG

Basil seed mucilage/gum was extracted following the methods described by Hosseini-Parvar et al. [20] and Rayegan et al. [21], with some modifications. The pH was adjusted to 7, and the temperature was maintained at 50 °C. The seed mixture was blended, and a cheesecloth was used to remove large seed debris. Fine seed particles were removed by centrifuging the mixture at 13,000× *g* for 30 min. The gum layer was then freeze-dried for further analysis.

BSG-WSE was extracted at 50 °C using an aqueous medium adjusted to pH 8 (8.0 ± 0.2) with 0.1 M NaOH at a seed-to-water ratio of 1:50 (*w*/*w*). The cleaned seed–water mixture was stirred with an overhead stirrer for 3 h at 100 rpm. The water-soluble polysaccharide layer was separated from the seed coat using a cheesecloth. The mixture was then stored overnight at 4 °C to allow sedimentation of starch and other insoluble or swellable polysaccharides. After sedimentation, the mixture was centrifuged at 4000× *g* for 30 min. The supernatant was freeze-dried and designated as BSG-WSE, with a yield of 2.25% based on seed weight. The extraction procedure was repeated twice. The resulting freeze-dried powder was subsequently mixed with protein candidates (NaCas and WPI) at different ratios for naringenin encapsulation.

#### 2.2.2. Composition Analysis

BSG-WSE was analyzed to determine crude protein (AOAC method 968.06), fat (AOAC method 922.06), ash content (AOAC method 942.05), and calcium (AOAC method 968.08D) [22]. Samples were pre-dried to constant weight.

#### 2.2.3. Total Uronic Acid Analysis

Total uronic acid content was analyzed for both crude BSG-WSE and alcohol-purified BSG-WSE using the m-hydroxyphenyl method [23] with a 96-well microplate reader (R^2 ^= 0.97%). The values are presented in terms of glucuronic acid.

#### 2.2.4. Acid Dissociation Constant (pK_a_) of the Polysaccharide-Rich BSG-WSE

To determine the pKa of crude BSG-WSE, a 0.125% (*w*/*v*) BSG-WSE solution was titrated with 0.1 M NaOH at 20 °C using an automated titration method (Titrator—Excellence T7, Mettler Toledo, Columbus, OH). Before titration, the solution was acidified to pH 1 using 0.1 M HCl. The sample was titrated from pH 1 to 12, and the titration curve was plotted between pH and volume (mL at 1 mL/min). The equivalence point was determined by plotting the first derivative of the graph (∆pH/∆Vt) and finding the maximum ∆pH/∆Vt. The pKa was considered the half-equivalence point in the buffer region.

#### 2.2.5. Molecular Characteristics of BSG-WSE

The molecular characteristics of the water-soluble fraction of BSG were analyzed using size exclusion chromatography coupled with a multiangle light scattering detector (SEC-MALLS, LC-20AD Shimadzu Corporation, Kyoto, Japan). Three detectors were used: a differential refractive index detector (IRD-20A), UV/Vis spectroscopy (SPD-20A) set to 227 nm, and a DAWN 8+ multiangle laser scattering detector (LS) with a laser at 690 nm. Freeze-dried powders were analyzed using 0.1 M NaCl as the mobile phase in isocratic elution mode. Solvents were vacuum filtered through 0.22 µm polyvinylidene difluoride (PVDF) and 0.02 µm nanocellulose filters. Samples were filtered through 0.45 µm PVDF filters. Separation of molecular fractions was achieved using a guard column (G1) and an aqueous polymer base column: Shodex SB-806 HQ (Showa Denko, Tokyo, Japan). The UV/Vis spectrophotometer wavelength was set at 227 nm based on previous analysis of the supernatant. For alignment and normalization in Astra 1.6 (Wyatt Technology Corporation, Santa Barbara, CA, USA), BSA was used as the standard. The specific refractive index (dn/dc) was chosen as 0.185 mL/g. The flow rate was set at 1 mL/min, and the column oven temperature was 40 °C with the injection volume of 10 μL (sample concentration 5 mg/mL). For sample analysis, dn/dc was chosen as 0.147 according to previous literature.

#### 2.2.6. Preparation of BSG and Protein Mixtures to Encapsulate Naringenin

Different ratios (*w*/*w*) of freeze-dried BSG-WSE and NaCas (1:1, 1:3, and 1:5) were tested using different pH treatments (4, 5, and 7) combined with heat treatments (60 °C and 80 °C for 30 min) to analyze the physical stability, encapsulation efficiency, and loading capacity of naringenin at each treatment. Naringenin concentrations were maintained the same for each treatment (0.25 mg/mL). For the 1:1 ratio, both BSG-WSE and NaCas concentrations were 0.075%. For the 1:3 ratio, the concentrations were 0.075% and 0.225%, respectively, while for the 1:5 ratio, they were 0.075% and 0.375%, respectively.

Suspensions of naringenin were alkalized to enhance solubility and subsequently mixed immediately with the other two components (combination) according to the given ratios. Initially, a fresh stock solution of naringenin was prepared (0.25%), and the pH of the solution was carefully monitored (11.0 ± 0.5, 20 °C, 60–120 s) until a clear solution was obtained (it was wrapped with tin foil). The required amount was immediately pipetted out to mix with other constituents (the pH was read at 10.0 ± 0.3 after the addition). These ternary mixtures (constant volume was maintained, 20 mL) were acidified to the target pH (4, 5, or 7) by adding 0.1 M HCl to induce complex formation. Samples at each pH point were heated using a water bath while stirring continuously (heat treatment: 60 °C for 30 min). pH-treated and heat-treated samples are termed as ternary systems throughout the studies. The encapsulation efficiency and loading capacity were analyzed at each selected pH. For each pH adjustment, a total duration of around 20 min was allocated, and 50 μL of acid was added each time, waiting until the pH reading stabilized while stirring. Control samples (only with protein) were also carried out for the comparison and to evaluate the stabilizing effect exerted by BSG-WSE.

The sample condition that gave the highest encapsulation efficiency (biopolymer ratio 1:1 with pH 4) was further tested with heat treatment at 80 °C for 30 min instead of 60 °C (for both heat treatments, the same ramping rate was employed). Since the treatment condition with 60 °C showed the highest encapsulation efficiency, the same conditions were used to test the system with rutin, quercetin, and WPI. The biopolymer ratio 1:1 was further tested with pH treatment 4 and heat treatment 60 °C for 30 min, with 2% (*w*/*v* aqueous solution) glucono-δ-lactone (GDL) as the acidifier instead of HCl to analyze the effect of the acidifier. After analyzing encapsulation efficiency, selected samples were freeze-dried for further analysis (interactions and structural morphology). Samples were frozen at a shelf temperature of −43 °C, followed by primary drying at 0.2 mbar.

#### 2.2.7. Physical Stability Measurements

Physical stability was assessed using the Turbiscan Lab by analyzing treated ternary mixtures (BSG-WSE, NaCas, naringenin) at a 1:1 biopolymer ratio. Turbidity was quantified through transmitted light (T%) at 1 min, with higher EE% conditions (pH 4 and 60 °C heat treatment for 30 min) selected for stability testing. Stability over 24 h was evaluated via the Turbiscan Stability Index (TSI). Each 20 mL sample was equilibrated prior to scanning. Additionally, UV–Vis spectroscopy was employed to monitor naringenin stability.

#### 2.2.8. Encapsulation Efficiency (EE%) and Loading Capacity (LC%)

To analyze the EE% and LC%, ternary mixtures were first centrifuged at 4000× *g* for 20 min at 20 °C to remove any insoluble matter immediately after cooling the treated ternary mixtures. The supernatant was ultrafiltered with a 10 kDa molecular weight cutoff filter unit (Amicon Ultra, Burlington, MA, USA), and the permeate was used to analyze the unencapsulated/free amount. This solution was mixed with methanol and vortexed three times. The aqueous/methanol mixture was filtered through 0.2 µm nylon syringe filters. The unentrapped amount was measured using a high-performance liquid chromatography (HPLC) method. A C18 column (Luna, 5 µm C18 100 Å, Phenomenex, Torrance, CA, USA) was used to separate naringenin, and was detected at 288 nm in a UV detector at a column oven temperature of 40 °C. The flow rate was 1 mL/min, and gradient elution was performed. The mobile phase consisted of 0.1% aqueous formic acid and acetonitrile. The gradient was 10% B for 1 min, increasing linearly to 90% B over 10 min, held at 90% B for 1 min, then returned to 95% A for re-equilibration until 18 min. The injection volume was 10 μL. For rutin and quercetin, wavelengths of 247 nm and 370 nm, respectively, were employed. The values of EE and LC were calculated using the following formulas:

(1) $$Encapsulation\;efficiency=\frac{\left(initial\;naringenin\;used-naringenin\;in\;the\;permeate\right)\times 100}{\left(initial\;naringenin\;used\right)}$$

(2) $$Loading\;capacity=\frac{naringenin\;encapsulated\;\times \;100}{weight\;of\;the\;encapsulated\;material\;after\;freeze\;drying}$$

#### 2.2.9. Fourier-Transform Infrared Spectroscopy

The freeze-dried powders were analyzed using attenuated total reflectance Fourier-transform infrared spectroscopy: ATR-FTIR (Thermo Fisher Scientific Inc., Waltham, MA, USA). The spectra were recorded within the range of 400–4000 cm^−1^ using Omnic^TM^ Spectra Software (Version1.9). The results were normalized and averaged by Origin 2023 (Northampton, MA, USA).

#### 2.2.10. Structural Morphology

Scanning electron microscopy (SEM) was used to study the morphology of the lyophilized powder. Samples were mounted on aluminum stubs with double-sided tape. The samples were sputter-coated with approximately 100 nm of gold (Baltec SCD 050 sputter coater, Luebeck, Germany) and viewed under the microscope at an accelerating voltage of 20 kV. SEM images are presented with auto-generated metadata as provided by instrument software.

#### 2.2.11. Flow Behavior

Flow curves were obtained using a rheometer (Paar Physica MCR 302 Anton-Paar, Graz, Austria) in controlled shear rate (CSR) mode at 20.0 ± 0.1 °C using the double gap geometry (DG-26.7). The shear rate was decided so that it increases from 0.01 to 1000 s^−1^ with a log ramp time setting, starting from 30 s and ending at 2 s.

#### 2.2.12. X-Ray Diffraction (XRD) of the Lyophilized Powder

XRD analysis was performed using a benchtop powder X-ray diffraction instrument (Rigaku Miniflex PXRD, Rigaku, The Woodlands, TX, USA) at 20 °C. Cu Kα radiation (λ = 1.54059 Å) was used. Lyophilized samples were mounted on the Hampton double tape. Data collection was controlled by SmartLab Studio II software v2020 (Rigaku, The Woodlands, TX, USA), with background correction applied. Due to variable sample sizes in the cryo-loops, data were scaled to the same rise in the background caused by the beam-stop shadow. All samples were analyzed in the 2θ angle range of 5° to 60°.

#### 2.2.13. Zeta Potential and Particle Size of the Particles

Particle size and zeta potential were measured using a Malvern Zetasizer Nano (Malvern Instruments Ltd., Worcestershire, UK). After each treatment, samples were immediately cooled in an ice bath and analyzed for particle size and zeta potential to determine nano-sized particles. For pH 7 samples, a Mastersizer 2000 (Malvern Instruments Ltd., Malvern, UK) was additionally used to measure larger particles (microscale). Samples were diluted across a range of concentrations, and the half-diluted sample was selected for analysis. Instrument settings were automatically optimized based on sample scattering, with attenuation recorded as 7.

#### 2.2.14. Release Behavior at Different pH Values

To analyze the effect of pH on the release behavior of naringenin, the freeze-dried encapsulated powder (0.2% *w*/*v*) was dissolved in different pH buffer solutions (pH 3: simulated gastric fluid, pH 7: simulated intestinal fluid, pH 8: simulated intestinal fluid adjusted to pH 8). Buffers were prepared according to the INFOGEST method without adding enzymes or bile salts [24]. Samples were stirred for 2 h, and the release amount at each pH was measured using the HPLC method described above (Methods Section 2.2.8). Before analyzing the release amount, samples were filtered using 10 kDa MWCO filters (Amicon Ultra).

#### 2.2.15. Data Analysis

Data are presented as mean ± standard deviation for triplicates. SPSS IBM 27 software and Origin 2023 software were used for statistical analysis and graphical illustrations, respectively. For multiple mean comparisons, ANOVA was used with Tukey’s test. Data were considered significant at *p* < 0.05.

## 3. Results and Discussion

### 3.1. Characterization of BSG-WSE

#### 3.1.1. Proximate Composition of BSG-WSE

BSG-WSE was analyzed to determine its crude protein, fat, ash, and calcium content. Uronic acid (glucuronic acid) was determined using the m-hydroxyphenyl colorimetric method. Table 1 shows the proximate composition of BSG-WSE.

**Figure 1:**
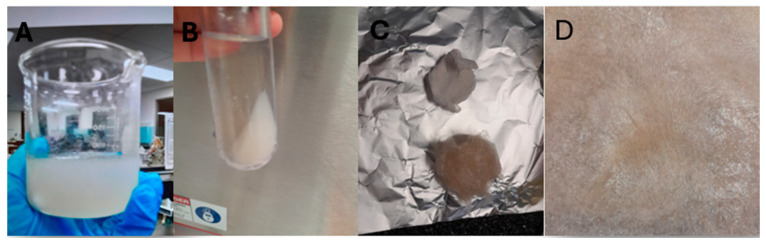
(**A**) Basil seeds gum/mucilage; (**B**) Ultracentrifuged basil seeds mucilage showing the supernatant with water-soluble polysaccharides; (**C**) Alcohol-purified basil seed gum water-soluble extract compared to crude sample; (**D**) Freeze-dried material of basil seeds gum water-soluble extract.

Figure 1A shows the basil seed gum/mucilage. The crude extract (BSG-WSE) showed a high glucuronic acid content (expressed as % of dry weight). BSG-WSE (Figure 1B–D) exhibited a considerably high amount of ash (inorganic material), of which ~1.3% was calcium. It also had high protein and uronic acid content. After alcohol washing, the glucuronic acid content significantly reduced to 7.62% ± 1.01%, whereas Naji-Tabasi et al. [25] reported a value of 13.39% ± 0.1% for SUPER-BSG obtained from the supernatant of gradual ethanol precipitation of BSG, which represented the low molecular weight fraction. In this study, BSG-WSE was washed with four volumes of absolute ethanol and then again with three volumes of ethanol, and the precipitate obtained was used for the uronic acid assay.

The method employed hydrolyzes polysaccharides with concentrated H_2_SO_4_, releasing uronic acid, which forms a colored complex with m-hydroxydiphenyl [26]. Previous studies reported significantly lower protein and ash content for the alcohol-purified “SUPER-BSG” [25]. Lower levels of glucuronic acid in a substance result in a reduced number of ionizable carboxylate groups, which in turn decreases the overall negative charge at neutral pH. This reduction in charge can lead to decreased electrostatic repulsion between particles and weaken the electrostatic attraction to positively charged protein domains, particularly near or below the protein’s isoelectric point. Consequently, the efficiency of protein–polysaccharide interactions may be affected, impacting processes such as complex formation and encapsulation. To compensate for the lower charge density, it may be necessary to adjust experimental conditions, including pH, ionic strength, or the amount of polysaccharide used, to achieve optimal protein–polysaccharide complexation and encapsulation performance.

**Figure 2:**
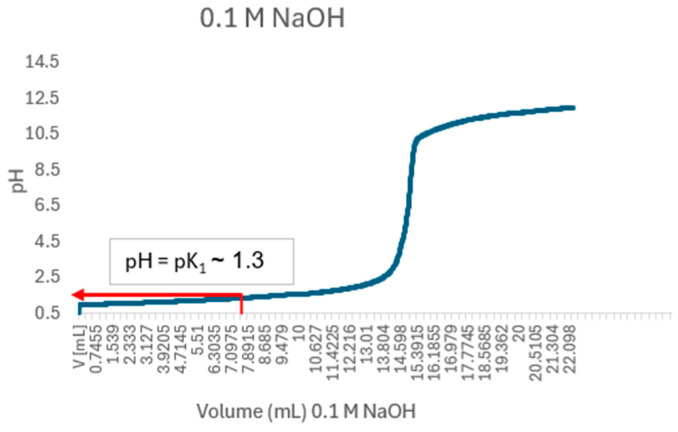
pKa value of basil seeds gum water-soluble extract (shown by the red arrow), representing dialyzed crude water-soluble polysaccharides. The first mid-point in the buffer region was considered (pK1) since it was a weak acid.

Figure 2 demonstrates the pKa value of basil seed gum water-soluble extract.

#### 3.1.2. Molecular Characteristics of BSG-WSE

**Figure 3:**
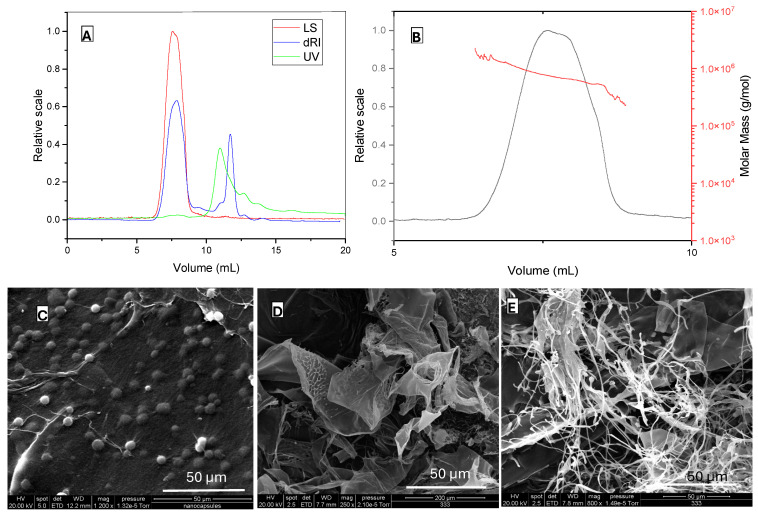
(**A**): SEC-MALS (size exclusion chromatography with multiangle light scattering) chromatogram showing LS (light scattering), dRI (refractive index) and UV (ultraviolet) signals in a relative scale, (**B**): Molar mass distribution of the polysaccharide fraction, (**C**): SEM (scanning electron microscopy) image of the basil seeds gum/mucilage with magnification ×1200, (**D**,**E**): SEM images of water-soluble polysaccharides of basil seeds gum (magnification ×250 and ×800).

The molecular characteristics of BSG-WSE are shown in Figure 3A–E. The emergence of a light scattering (LS) peak around the retention time of 7–9 min (Figure 3A) indicates the presence of polysaccharides. Molecular weight distribution is shown as a function of elution volume. Molecular weight was determined by the Zimm plot, with a weight-average molar mass of approximately 7.37 × 10^5^ Da and a polydispersity index of around 1.09 (Mw/Mn), indicating a more monodisperse polymer. Goh et al. [27] reported a similarly low value for the polydispersity index for chia seed polysaccharides. However, the molecular weight distribution plot (Figure 3B) suggests the presence of an overlapping peak, potentially merging with the primary peak. The root-mean-square radius was around 72.4 nm. It has been postulated that the exponent value of the curve obtained between the mean-square radius and molecular weight indicates the conformation of the polymer [28]. The theoretical values suggest an exponent value, 0.5 represents a random coil, while 0.6 represents a linear chain in a good solvent. Accordingly, our value of 0.55 ± 0.02 suggests that this polymer behaves as random coils in the solution (0.1 M NaCl).

An additional refractive index (RI) peak around the retention time of 11–12 min (Figure 3A) suggests the possibility of a fraction with a smaller molecular weight, such as proteins, due to its low absorbance in LS. Additionally, a UV peak at 227 nm detected using UV/Vis spectroscopy could represent proteins or phenolic compounds that may exist in the water-soluble extract either in free form or bound to polysaccharides. This UV peak was also visible in the ethanol-washed extract, suggesting they could be bound to polysaccharides. Low-resolution fractions eluting after 12 min likely correspond to sugars, polyphenols, and proteins. The absence of light scattering (LS) peaks in this region indicates that no high-molecular-weight polysaccharides or complexes were present. The simultaneous detection of refractive index (RI) and ultraviolet (UV) signals suggests the presence of smaller molecules. The UV peak near 227 nm may be attributed to aromatic amino acids or other UV-active functional groups.

Figure 3C–E shows SEM images of BSG and BSG-WSE. Figure 3C depicts the basil seed gum mucilage with a network-like structure composed of cellulose threads and starch granules [29]. In terms of BSG-WSE, SEM analysis revealed various types of polysaccharides, including thread-like structures that were intertwined to form fibrous networks and flat, ribbon-like formations as observed in Figure 3D,E. Helical arrangements were also visible at higher magnifications. These structural differences could be attributed to their monosaccharide composition, molecular weight, and uronic acid composition [30]. Additionally, some globular structures attached to the water-soluble fraction could represent glycoproteins.

### 3.2. Encapsulation Efficiency and Loading Capacity

#### 3.2.1. Effect of Mixing Ratio, pH, and Temperature

The interaction between proteins and polysaccharides is complex and influenced by factors such as pH, salt concentration, biopolymer ratio, polyphenol concentration, and temperature. In this study, different biopolymer ratios were tested under various pH conditions to encapsulate a fixed concentration of naringenin. The objective was to evaluate how each variable affects encapsulation efficiency and to identify strategies for optimizing protein–polysaccharide complex formation for improved delivery systems.

**Figure 4:**
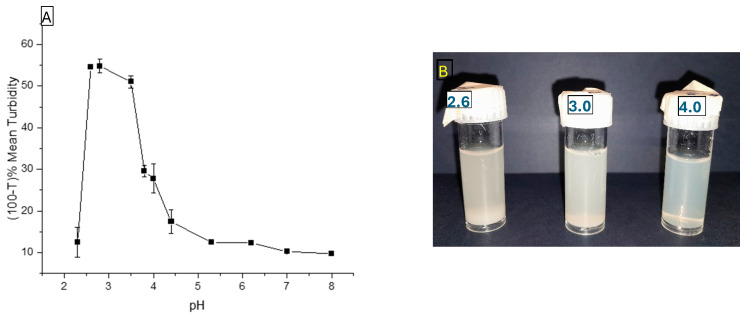
(**A**): Turbidity variation at different pH points when decreasing the pH from alkaline to acidic conditions. (**B**): Ternary mixtures of the basil seed gum water-soluble extract, sodium caseinate (1:1 ratio), and naringenin at different pH treatments only.

To analyze the effect of pH on maximum complex formation, turbidity measurements were recorded at each point across the following pH range (Figure 4A), using a 1:1 biopolymer ratio (NaCas to BSG-WSE) in the presence of naringenin. Turbidity and stability measurements were conducted using Turbiscan Lab, which uses a near-infrared LED (λ_air_ = 880 nm) as the light source. Accordingly, the maximum coacervation yield was obtained at pH 2.6 for the BSG-WSE and NaCas combination in the presence of naringenin (concentration: 0.25 mg/mL). Soluble complex formation (pH_c_) starts at pH 5, and pH_opt_ was shown at pH 2.6 (Figure 4A), and these values were determined by locating the onset of turbidity increase and its maximum, respectively. Figure 4B illustrates the visible turbidity of the ternary mixtures (pH treated only).

Since pH 2.6 is too low for food applications and showed sedimentation behavior, the treatment condition at pH 4 was chosen for synthesizing the encapsulation system, and heat treatment was employed to reinforce the interactions as the second step. The turbidity of the heated sample (60 °C for 30 min) was 34.6 ± 1.5%, corresponding to 100–T% at 880 nm, and the non-heated sample was 17.4 ± 2.8%. This represents an increase of nearly 17% in turbidity compared to the non-heated sample, suggesting more interactions. To further understand the system’s behavior, encapsulation efficiencies at different biopolymer ratios at both neutral and acidic pH values were tested with the heat treatment.

**Figure 5:**
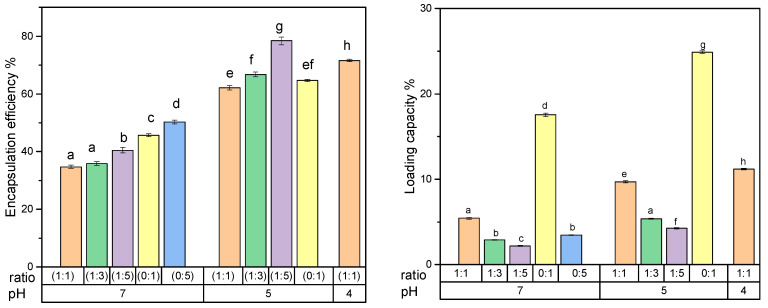
Encapsulation efficiency and loading capacity at different pH values at different basil seeds gum water-soluble extract to sodium caseinate ratios. Different letters indicate statistically significant differences (*p* < 0.05), *n* = 3.

Figure 5 illustrates the impact of different pH values and biopolymer ratios on the encapsulation efficiency and loading capacity of naringenin when creating stable colloidal systems without sedimentation. The highest encapsulation efficiency was observed at pH 5 for a biopolymer ratio of 1:5 (total biopolymer concentration of 0.45% *w*/*v*). However, this ratio did not yield the highest loading capacity. It was also noted that higher total protein concentration resulted in higher encapsulation efficiency, indicating that naringenin is more attracted to proteins. This was further confirmed by the controlled samples carried out. Herein, the influence of total concentration should not be underestimated. However, the controlled samples at pH 5 showed the initial signs of sedimentation (the particle size ranged between 20–30 µm, resulting in a visibly gritty appearance), while the controlled sample at pH 4 was completely precipitated. According to Figure 5, for a given polymer ratio, the highest colloidally stable interactions and the highest loading capacity were observed at pH 4 for a 1:1 polymer ratio, attributed to the charge distribution of the mixture. At a specific pH level, the ionization of functional groups alters the charge distribution, thereby modulating repulsive or attractive forces within the matrix. These Coulomb-driven interactions govern the self-assembly and shape of the colloidal particles. This is also one of the factors that governs the stability of the system. The viscosity effect exerted by biopolymers of the system also plays a critical role in preventing sedimentation. The dialyzed crude BSG-WSE has an apparent pKa of 1.37 (Figure 2), as determined through titration. This low pKa indicates that its acidic groups are nearly fully deprotonated at pH levels above approximately 2. As a result, the polysaccharide fraction remains strongly negatively charged (anionic) across the entire experimental pH range. This persistent negative charge facilitates electrostatic interactions primarily with proteins that carry a positive charge, which occurs between pH 2 and the protein’s isoelectric point (pI ≈ 4.6). At this pH, the protein has a net positive charge, promoting strong electrostatic attraction between the polysaccharide and the protein. Additionally, the notably low pKa value may suggest that the carboxylic groups within the polysaccharide are either overlapping or closely spaced, which could influence their ionization behavior and interaction potential within the system. Understanding these electrostatic interactions is crucial for elucidating the behavior of the ternary system, especially in applications involving protein-polysaccharide complexes or formulations where charge interactions play a significant role. The pKa of naringenin, reported as 7.86, 9.20, and 9.79 [10], should also be considered in this system.

**Figure 6:**
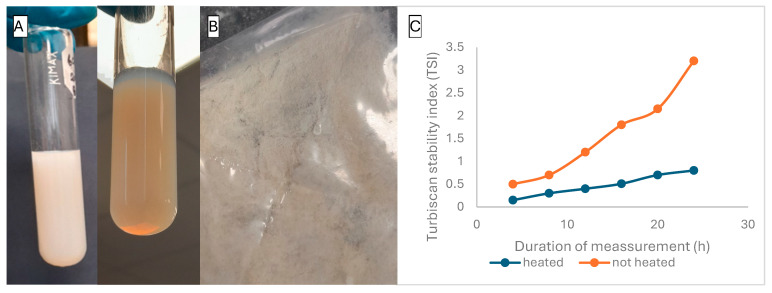
(**A**): Ternary system composed of basil seed gum water-soluble extract, sodium caseinate, and naringenin at pH 4, acidified with 2% glucono-δ-lactone prior to freeze-drying. (**B**): Freeze-dried ternary system. (**C**): Stability index over 24 h for heated (blue) and non-heated (orange) systems.

For the control naringenin sample (without BSG-WSE or NaCas), naringenin sedimented at pH 6.7 upon pH reduction from alkaline to acidic. Heat treatment increased the solubility of naringenin, but it sedimented again upon cooling, indicating temperature-dependent solubility and limited colloidal stability. The ternary system (BSG-WSE, NaCas, and naringenin) showed no spontaneous sedimentation at pH 4 for the 1:1 biopolymer ratio (Figure 6A). Upon forced centrifugation at 12,000× *g* for 20 min, insoluble particles precipitated at the bottom of the centrifuge tube, indicating the beginning of a complex coacervate system. The presence of anionic polysaccharides in the BSG-WSE extract contributes to system stability through charge distribution and the formation of a network-like structure (Figure 6B) at the given pH (total biopolymer concentration of 0.15% *w*/*v*).

Heat treatment further increased the stability of the system, as indicated by Turbiscan values (TSI values: 0.7 for heated and 2.5 for non-heated) over 24 h (Figure 6C). Protein controls showed sedimentation for all concentrations (except for the 0:1 ratio at pH 5) at both pH 4 and 5 after both treatments, indicating protein denaturation. However, protein controls had higher encapsulation efficiency compared to mixed counterparts, indicating naringenin’s higher affinity towards the protein candidate [31]. The addition of polysaccharides stabilizes the system without creating larger particles. Additionally, preliminary studies showed that basil seed mucilage acts as an adsorption mat-like structure for flavonoids at pH 7. Apart from this, the inclusion of polysaccharides also provides mechanical strength and shields polyphenols and proteins.

The system protects encapsulated compounds from oxidative damage and enzymatic degradation, enabling controlled release. Although the binary system initially entraps a larger proportion of compounds, it exhibits lower stability compared to the ternary system, as evidenced by sedimentation after overnight chilling. This instability suggests that a significant portion of the encapsulated material may be lost during storage or digestion. Conversely, the ternary system demonstrates superior colloidal stability, making it more suitable for incorporation into food models and enhancing its practical applicability. Additionally, the ternary system has a higher loading capacity, as shown in Figure 5, which reduces ingredient requirements and facilitates scalability. Overall, despite its lower initial encapsulation efficiency, the ternary system provides a better balance between stability, loading capacity, and potential for application.

Figure 6A illustrates the coacervation system formed at pH 4 using 2% GDL in place of 0.1 M HCl, shown with light illumination (right) and without illumination (left). According to our experiment, both samples treated with HCl and GDL had similar encapsulation efficiencies. This could be due to the slow addition of 0.1 M HCl (50 μL at a time, over approximately 20 min until the desired pH was reached) to the mixture, facilitating a gradual decrease in pH. The stability of the systems was further measured using zeta potential and size measurements. The size showed a significant decrease compared to the HCl counterpart (Table 2). The study conducted by Wang et al. [32] also reported on the previous studies that followed a similar trend when using GDL. Zhang et al. [33] reported that using HCl as the acidifier causes uneven particle size due to local precipitation, prolonging the acidification time, whereas GDL results in a more uniform size with a narrow distribution.

Regarding the effect of heat treatment at 80 °C for 30 min, the mixture showed an encapsulation efficiency of around 67%, which is lower than the heat treatment at 60 °C, and the particle size was smaller (322.7 ± 4.059 nm) compared to the treatment at 60 °C. A similar trend was observed by Wu et al. [34] for soy protein isolate and carboxymethyl cellulose combination. This particular treatment may have denatured the protein structure, resulting in smaller particles. Depending on the applied temperature conditions, protein denaturation can be either reversible or irreversible. Higher temperatures generally lead to a greater degree of irreversibility. It has previously been proposed that the disulfide bonds stabilizing milk protein structures work alongside hydrophobic interactions to facilitate protein aggregation during heat treatment.

Previous research has suggested that disulfide bonds, which stabilize the structure of milk proteins, coexist with hydrophobic interactions to facilitate protein aggregation during heat treatments. When disulfide bonds in whey protein isolate (WPI) are disrupted, the temperature at which aggregation begins with gum Arabic decreases significantly. This indicates that disulfide bonds play a role in limiting excessive aggregation. This understanding provides a potential mechanistic explanation for the observation that milder heat treatments lead to the formation of colloidally stable particles that are larger and have a greater capacity for encapsulation. Under these conditions, disulfide bonds may help maintain certain aspects of the protein’s tertiary structure, thereby enabling more effective entrapment of compounds such as naringenin within protein–polysaccharide complexes. In contrast, higher temperature treatments (e.g., 80 °C for 30 min under acidic conditions) can induce irreversible unfolding of WPI. Such unfolding exposes additional thiol groups, increasing opportunities for both covalent and non-covalent interactions with polysaccharides and naringenin. This promotes tighter complex formation and results in smaller particle sizes. However, during this structural rearrangement, competitive binding between naringenin and polysaccharides may lead to partial release of naringenin back into the solution, which likely explains the reduced encapsulation capacity observed at 80 °C.

Table 2 presents the corresponding variations in particle size, polydispersity index (PDI), and zeta potential across different pH values and mixing ratios. Based on these findings, the milder heat treatment condition was selected for subsequent studies.

When considering the effect of pH, at pH 7, the size of the particles was measured using the Mastersizer since samples at pH 7 showed micron-sized particles (D[4,3] values were considered and values are presented in nm). Initially, the sample size was measured using the Zetasizer. Since the observed size exceeded 1000 nm, the sample was subsequently analyzed using the Mastersizer to detect the presence of larger particles, if any. Accordingly, particle size at pH 7 was determined using laser diffraction (Mastersizer), whereas samples at pH 4 and 5 were analyzed by dynamic light scattering (Zetasizer). At pH values above the pI of proteins, polysaccharide and protein interactions occur mainly due to hydrophobic interactions, although electrostatic bonds may also form between positive patches of proteins and polysaccharides [35]. This may produce relatively large gel particles, in which naringenin could be entrapped via hydrophobic bonds. Naringenin could act as a cross-linking agent. In terms of biopolymer ratio, higher protein content resulted in comparatively smaller particles, likely due to increased interactions between protein and polyphenols, tightening the protein structure and overall gel particle size.

As seen in the zeta potential variation, higher protein content shields the negative charges in polysaccharides, resulting in lower negative zeta potential. The mild treatment applied likely did not induce any aggregations at this particular pH, which could be another reason for the comparatively smaller particle size. At pH 5, relatively smaller particles were formed, likely due to the formation of soluble complexes, as proven by the turbidity curve as well. However, the system behaved differently at a 1:3 ratio compared to pH 7. However, at pH 7, the effect of increasing the concentration of BSG-WSE on encapsulation was not analyzed. It was assumed that viscosity at that level renders the system sufficiently flexible to allow molecular motion towards binding sites.

Samples at pH 4 showed significantly larger particle sizes, confirming higher interactions with polyphenols and polysaccharides. The presence of insoluble particles upon centrifugation confirms the inception of a coacervation system. Increased turbidity, higher encapsulation efficiency, and loading capacity further support this evidence. Heat treatment increased turbidity, showing enhanced interactions after the heat treatment, a trend followed in size measurements, resulting in larger particles after the temperature treatment. According to TSI values, heating increased the stability of the system (Figure 6C) at pH 4, possibly due to restructuring of the system in a way that negatively charged polysaccharides coat the proteins to increase steric hindrance. This was further evident by comparing heated and non-heated samples, where the zeta potential values were −32.98 ± 0.28 mV and −28.92 ± 0.81 mV, respectively (pH values were measured at comparable ionic strength). This phenomenon may prevent further aggregation and increase the stability of the system. Similar behaviour was observed in previous studies [36]. Babu et al. [37] suggest that this effect is temperature and pH-specific, where in some instances, heat treatment results in a larger particle size and high encapsulation efficiency, but in other instances, they behave differently. A similar trend was observed in our study, with comparatively lower encapsulation efficiency and lower particle size at 80 °C than at 60 °C.

At a pH of 4, the samples demonstrated a relatively high polydispersity index (PDI approximately 0.5), indicating a broad range of particle sizes and limited uniformity within the sample. This level of heterogeneity suggests that the particles are more accurately described as submicron particles rather than a uniform population of nanoparticles. From an application standpoint in the food industry, submicron particles are generally considered safer and offer several practical benefits. These include enhanced colloidal stability, a lower tendency to aggregate due to their reduced specific surface area, easier scalability during production, and the potential for controlled or sustained release of encapsulated ingredients. Understanding these characteristics is crucial for optimizing formulation stability and ensuring safety in food applications, where particle size distribution can significantly influence product performance and consumer safety.

When pH 4 samples were kept overnight in the refrigerator, naringenin that had not been entrapped sedimented at the bottom of the sample container due to recrystallization (the sedimented amount was also analyzed and correlated with the encapsulation efficiency). Furthermore, wall material (only BSG-WSE and NaCas) without naringenin showed a lower particle size with higher zeta potential, confirming the encapsulation of naringenin in ternary mixtures. Further decreasing the pH (from 4 to 3) of the wall material resulted in a decrease in the negative zeta potential down to −13.4 ± 0.304 mV and a large particle size (341.23 ± 7.47 nm). At pH 4, other biopolymer ratios precipitated.

#### 3.2.2. Effect of WPI on the Encapsulation System

In this experiment, the impact of another protein candidate was also tested using whey protein isolates as the protein candidate. Accordingly, the encapsulation efficiency is lower for WPI, 66.76 ± 4.8% when compared to the sodium caseinate counterpart, which was 71.55 ± 0.77%, at pH 4 for a 1:1 protein to polysaccharide ratio (*p* < 0.05). A similar trend was indicated by Yin et al. [38] for resveratrol, where both NaCas and WPI were used to compare the encapsulation efficiency. NaCas is structurally more hydrophobic than WPI, and this could be a plausible reason for the high encapsulation efficiency of NaCas. Furthermore, the disordered structure of NaCas may tend to encapsulate more naringenin than WPI with two major globular proteins, namely β-lactoglobulin and α-lactalbumin [39]. When considering the molecular weight of these proteins, it is much higher for NaCas, and this could also be a contributing factor to the higher encapsulation efficiency of NaCas. However, Mao et al. [40] stated that the effect of treatment conditions should also not be overlooked, especially the pH treatment, ionic strength, and polyphenol concentration. In this study, only one pH (pH 4) was tested. There could be an effect from the isoelectric point of these proteins, as their values slightly differ; it may influence the net charge of the system and affect the bond formation.

#### 3.2.3. Comparison with Other Flavonoids

Instead of naringenin, the system was tested for quercetin and rutin using the biopolymer ratio (1:1) at pH 4 and 60 °C. Interestingly, quercetin exhibited the highest encapsulation efficiency (94.36 ± 5.81%) and loading capacity (14.1% ± 3.12%) among the tested flavonoids, while rutin showed the lowest values (32.36% ± 4.29%). Compared with naringenin, quercetin contains additional hydroxyl groups that provide more potential interaction sites with both proteins and polysaccharides. Quercetin’s 3′-hydroxyl group on the B-ring carries a relatively higher negative charge, and its planar conformation, arising from the C2=C3 double bond in the C-ring, enhances its capacity for hydrogen bonding, hydrophobic interactions, and particularly π–π stacking [41]. By contrast, naringenin is a flavanone with a saturated C-ring and three hydroxyl groups, resulting in moderate polarity but overall greater hydrophobicity. Its less planar structure favors hydrophobic interactions rather than extensive π–π stacking. As observed by Tu et al. [42], naringenin tends to exhibit groove-type binding to DNA, inserting into the minor groove rather than intercalating broadly into the double helix. This site-specific binding preference suggests that naringenin interacts with a limited number of structural motifs rather than distributing uniformly across a carrier matrix, which may contribute to its comparatively lower encapsulation efficiency. However, while this structure-function relationship may explain the plausible mechanism underlying the ternary system, it needs to be supported by experimental evidence.

In protein systems, naringenin typically binds within hydrophobic pockets, where Van der Waals forces and hydrogen bonding stabilize the interaction. This localized binding can induce subtle conformational tightening of the protein structure, potentially reducing the availability of additional binding sites and limiting overall loading capacity. However, these selective, high-affinity interactions can enable controlled or stimulus-responsive release, as the bound naringenin becomes more sensitive to environmental factors such as pH and ionic strength [43]. In contrast, rutin showed noticeably lower binding affinity toward the protein–polysaccharide complexes. The attached sugar moiety introduces steric hindrance, reducing the molecule’s ability to closely approach and interact with protein or polysaccharide surfaces. Because the sugar group is positioned at the 3′-hydroxyl site on the B-ring, an important locus for hydrogen bonding, it effectively blocks one of the key interaction sites. Additionally, the hydrophilic nature of the sugar moiety reduces hydrophobic association compared with naringenin. Competition for binding between rutin’s glycosyl group and the polysaccharide components (such as those in BSG–WSE) further limits its ability to interact effectively with NaCas.

### 3.3. Possible Interactions in Encapsulation Systems

After analyzing the results of encapsulation efficiency, size, zeta potential, and stability data, the pH 4 sample at a 1:1 BSG-WSE to NaCas biopolymer ratio was selected for further analysis of structural properties and interactions.

To analyze the structural properties of ternary complexes, FTIR-ATR, XRD, and microscopic techniques (e.g., SEM) were performed on freeze-dried samples. IR spectra of both individual components and different mixed systems were obtained to analyze possible interactions that may form between components.

**Figure 7:**
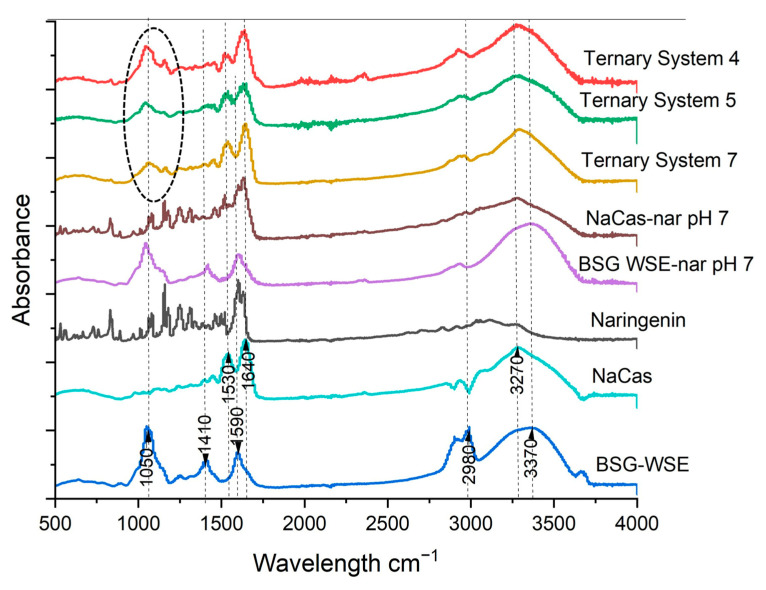
FTIR spectra of individual components (BSG-WSE; basil seeds gum water-soluble extract, NaCas: sodium caseinate and naringenin) and some of the ternary systems at different pH values.

Figure 7 demonstrates the FTIR spectra of individual components (lower portion of the graph, with major functional groups labeled) and some of the ternary complexes (biopolymer ratio 1:1) at different pH values (upper part), depicting notable differences in interactions. For BSG-WSE, hydroxyl groups (including inter- and intramolecular hydrogen bonds) are denoted by the peak around 3370 cm^−1^. The peak around 1050 cm^−1^ in the fingerprint area indicates the typical O-C-C stretch in the glycosidic bond of polysaccharides. Additionally, the peaks around 1590 cm^−1^, 1410, and 638 cm^−1^ may represent the glucuronic acid, which has been reported to be present in BSG [44]. The table (Table 3) also illustrates the possible functional groups present in the BSG-WSE. The glucuronic acid assay conducted in this study also showed that BSG-WSE possesses a high amount of glucuronic acid.

The peaks corresponding to prominent functional groups in NaCas are depicted in Figure 7. Amide I and II bonds are represented by the peaks around 1600 cm^−1^ and 1530 cm^−1^, respectively. The peak at 3270 cm^−1^ in the NaCas spectra depicts the NH bond in NaCas. When considering the sample at pH 4 (1:1 ratio) and compared to the NaCas control, a red shift occurs in the peak corresponding to CH_2_ stretching (peaks window 2800 cm^−1^–3000 cm^−1^, 10 cm^−1^ red shift from 2980 cm^−1^), indicating increased interactions or electrostatic attractions. The red shift in the CH_2_ region suggests a possibility of weakening of the C-H covalent bond and formation of hydrogen bonds, which cause the bond to vibrate at a lower frequency.

Furthermore, complex formation and increased molecular weight contribute to a lower frequency. Since this region represents the nonpolar area of the molecule, the red shift also demonstrates the possibility of the formation of hydrophobic interactions. Nevertheless, changes in the local environment and overlapping bands, rather than specific interactions, may also lead to redshifts. Furthermore, the emergence of peaks around 1050 cm^−1^ in the pH 4 sample, which may correspond to C-O-C either bonds or C-O bonds in hydroxyl groups, suggests the earlier hypothesis discussed under zeta potential: the possibility of a higher contribution of the polysaccharide at the surface around naringenin-bound protein particles. The peak area increased when lowering the pH, which aligns with the zeta potential data discussed earlier (the peak area is shown with a dashed circle). The spectral signals of proteins appear to be overwhelmed by the more dominant polysaccharide peaks.

In terms of the amide I peak in NaCas, peak broadening was observed in ternary mixtures, more prominently at pH 4 and 5. When considering the NaCas-naringenin pH 7 control (middle of Figure 7), this effect is clearly visible, indicating increased interactions. At pH 7, this could be due to hydrophobic interactions and/or hydrogen bonds. Broadening of the NH_2_ peak in the NaCas-naringenin pH 7 sample suggests possible H-bond formation. In this control sample, most of the NaCas peaks in the window of 1500 cm^−1^–500 cm^−1^ have been overlapped by peaks of naringenin. For the ternary mixture (at pH 4 and 5), this window could indicate increased electrostatic attractions between protein and polysaccharides too. Considering the BSG-WSE-naringenin control (encapsulation efficiency: 25.38% ± 0.48%), the peak around OH (3370 cm^−1^) increased, suggesting increased intermolecular H bonds. Similar results were obtained by Guo et al. [49], who studied the ability of corn silk polysaccharides to bind flavonoids. However, the peak broadening and overlap in the 1500–500 cm^−1^ region of the FTIR spectrum limit exact functional group assignments and, therefore, complementary techniques such as NMR (Nuclear Magnetic Resonance), Raman spectroscopy, or model binary systems are often required.

The interactions that may form among these compounds include covalent and non-covalent bonds such as hydrophobic interactions, hydrogen bonds, van der Waals interactions, and electrostatic interactions. Among these, hydrogen bonds are the most common bonds between protein and polyphenols, and these binary compounds have been proven to enhance the bioavailability, stability, and prevent the photolysis process, thereby increasing the therapeutic effects of polyphenols. Apart from hydrogen bonds, the temperature treatment applied in the process could also enhance the hydrophobic bonds due to the enthalpic effect. When combined with polysaccharides, the ternary system also creates similar non-covalent bonds. The reversible nature of non-covalent bonds makes the system conducive to stimulus-triggered release.

Additionally, the alkaline treatment involved in the process may create covalent bonds with protein and polysaccharides through the formation of semiquinones and quinones, which act as strong nucleophiles [50,51,52]. Polyphenols can undergo oxidation to quinones under alkaline conditions. These quinones are electrophilic and can react with nucleophilic groups on proteins, particularly amino (–NH_2_) and thiol (–SH) residues, leading to the formation of protein-quinone adducts. These adducts may subsequently crosslink with polysaccharides, generating covalent interpolymer networks. Such interactions are supported by the FTIR data. The broadening of the –OH stretching band in the pH 4 sample (red spectrum) is consistent with enhanced hydrogen bonding, but it may also reflect covalent bond formation involving phenolic hydroxyl groups. Moreover, the appearance of a new band in the 1000–1200 cm^−1^ region in the pH 4 sample, absent in the polysaccharide control, indicates structural modifications within the polysaccharide backbone, likely arising from covalent crosslinking.

Alkalization is often necessary to solubilize flavonoids; however, it can inadvertently promote quinone formation and subsequent covalent coupling. These reactions may alter the chemical structure of proteins and polysaccharides, potentially affecting their nutritional and functional properties. To better understand these modifications, future studies should employ advanced analytical techniques such as liquid chromatography–mass spectrometry to accurately quantify structural changes. Such analysis is essential for evaluating the impact of these alterations on biomolecular functionality. Additionally, the physiological implications of quinone intermediates warrant further investigation, as they can exhibit reactive or undesirable biological effects. Nonetheless, rapid alkalization followed by encapsulation within a polymeric matrix may help minimize these effects by stabilizing reactive intermediates and limiting their free reactivity in solutions.

**Figure 8:**
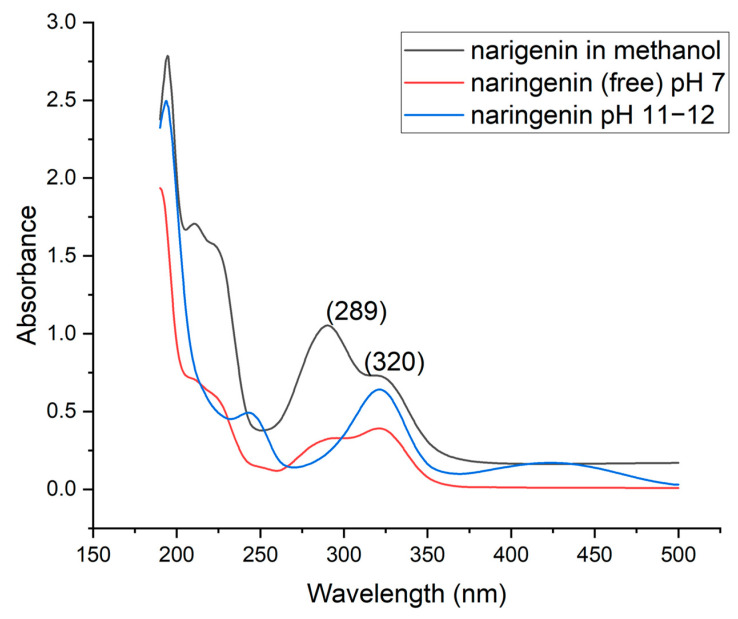
UV-–Vis (ultraviolet–visible) spectrum of naringenin showing the characteristic peaks corresponding to A ring (289 nm) and B ring/phenolic part (320 nm) in methanol, compared with alkaline naringenin (pH 11–12) and unencapsulated/free naringenin at pH 7 in aqueous medium.

The UV–Vis spectrum of pure naringenin, as shown in Figure 8, displays the characteristic peaks associated with this compound, confirming its typical spectral profile [53]. When the sample is treated with an alkaline solution, represented by the blue line, an additional peak appears around 250 nm and another near 420 nm. These new peaks suggest the formation of possible adducts or chemical alterations that occur during the alkalization process. Such changes may be reversible or irreversible, depending on subsequent acidification steps. The red line illustrates the spectrum of unencapsulated naringenin at pH 7, which retains the characteristic peaks, indicating that the experimental procedures employed had minimal impact on the structural integrity of naringenin. Overall, these spectral observations provide insights into the chemical stability and potential modifications of naringenin under different pH conditions, which are critical for understanding its behavior in various applications.

### 3.4. Powder X-Ray Diffraction Patterns

**Figure 9:**
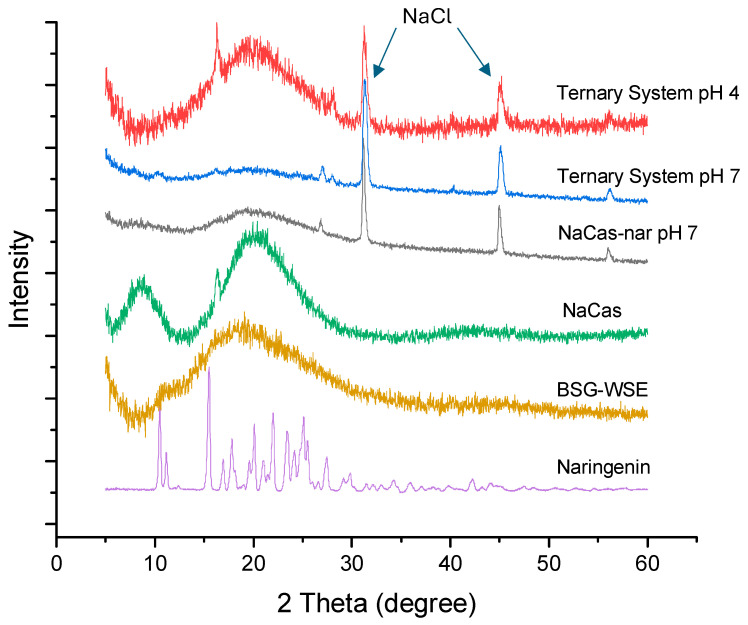
X-ray diffraction patterns of freeze-dried powders. BSG-WSE: basil seeds gum water-soluble extract, NaCas: sodium caseinate, Ternary system: naringenin with BSG-WSE and NaCas.

Figure 9 depicts X-ray diffraction patterns for individual components and the ternary complex. The treatment conditions have rendered naringenin amorphous, thereby facilitating its water solubility. When naringenin crystals are alkalized, they dissociate into their deprotonated form, enhancing the solubility of the powder based on their pKa values. Subsequently, mixing this alkalized naringenin solution with both polysaccharide and protein disperses the naringenin molecules, facilitating interactions with biopolymers upon subsequent acidification. Therefore, naringenin exists in an amorphous form.

Acidification with HCl inevitably results in residual sodium chloride (NaCl) in the mixture, and small peaks appearing in the powder X-ray patterns (Figure 9) could stem from NaCl crystals deposited on the surface. SEM images also demonstrated salt crystals deposited on the surface.

**Figure 10:**
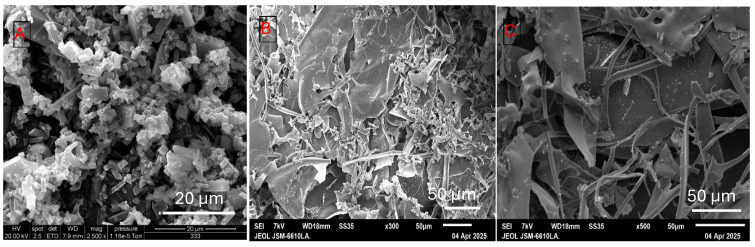
(**A**): Naringenin control, magnification ×2500, (**B**,**C**): Binary mixture of sodium caseinate and naringenin at acidic pH (pH 5), at magnifications of ×300 and ×500.

Sharp, well-defined peaks were observed for naringenin, and similar diffraction patterns were observed in other studies as well [54,55]. Considering the pH 7 sample, the ternary mixture exists as an amorphous mixture, giving a broader diffusive peak. At pH 7, protein may partially unfold and may electrostatically bind with polysaccharides, preventing the aggregation of protein and creating a mesh-like structure accommodating naringenin on the surface and inside the ternary structure. SEM images, further supporting this hypothesis (Figure 10B), demonstrated naringenin crystals dispersed on the BSG-WSE and NaCas mat-like structure. Apparently, the treatment conditions applied turned naringenin into small crystal domains (grain boundaries created disrupt the uniformity of the crystals) and hence, most of the sharp crystalline peaks shown by naringenin control disappeared in the ternary mixture. In contrast, macro salt crystals formed on the surface due to the neutralization process were depicted by sharp peaks. These peaks, which resulted from the neutralisation process between NaOH and HCl because of the pH treatment carried out in the procedure, do not overlap with the peaks of naringenin. Similar XRD patterns were observed by Rashidinejad et al. [56] for rutin, NaCas, and trehalose mixture.

The pH 4 mixture also demonstrates an amorphous nature, which is represented by a broader diffused peak starting from the diffraction angle 10° to 30°. Compared to NaCas and BSG-WSE, the peak positioning of the ternary mixture at pH 4 is different, and almost all the sharp peaks shown by naringenin have disappeared, suggesting the formation of small domain crystals of naringenin.

The amorphous nature of the surrounding material prevents recrystallization of naringenin during storage. Added to this, the amorphous nature and the network-like structure of the wall material are also contributing factors for the high encapsulation efficiency owing to its high surface area. An amorphous encapsulation system generally doesn’t compromise the rheological characteristics of a food system, such as pseudoplastic behavior, which is highly compatible within a food system. Moreover, during the digestion process, an amorphous nature helps release the entrapped bioactive compounds because of its high surface area and solubility.

### 3.5. Morphology of Ternary Mixtures

SEM micrographs of the encapsulated systems show that naringenin is well dispersed within the polymer matrix. Figure 10A illustrates the surface morphology of control naringenin, while Figure 10B,C depicts binary mixtures of protein and polyphenols. In the control sample (Figure 10A), the treatment produced large, aggregated crystals, indicating that the conditions alone were insufficient to generate smaller crystals that could enhance naringenin’s solubility. Additionally, salt crystals were observed in the treated sample.

SEM images of the binary mixture reveal random sheet-like structures with aggregated or denatured proteins, along with small polycrystalline naringenin crystals dispersed on the surface. Additional analyses, such as staining or Energy-Dispersive X-ray Spectroscopy (EDS), are needed to confirm these observations. The pH shift from alkaline to acidic conditions promotes protein aggregation, and the presence of naringenin may accelerate precipitation, contributing to the aggregated structures seen in Figure 10B. As evident in the SEM images, hydrophobic and hydrogen bonds may form between aromatic rings of polyphenols and aliphatic or aromatic rings of proteins. These interactions alter the surface structure, reducing its hydrophobicity.

These individual crystalline grains of naringenin offer a high surface area to contact with the solvent and hence increase the solubility of naringenin. Formation of different bonds alters the surface structure, making it less hydrophobic. However, at lower pH levels, proteins tend to be precipitated, losing their thin sheet-like structure. Hence, the incorporation of BSG-WSE hinders the aggregation of proteins while offering a high surface area of wall material to entrap more naringenin.

**Figure 11:**
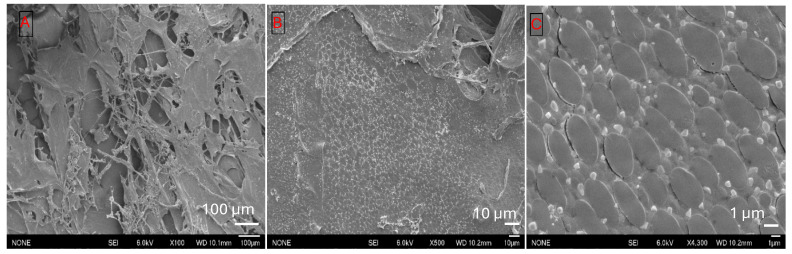
Ternary systems (basil seeds gum water-soluble extract, sodium caseinate, and naringenin) at pH 7 at different magnifications ((**A**): ×100, (**B**): ×500, and (**C**): ×4300).

Figure 11 illustrates the encapsulation system at pH 7. Unlike the protein control (Figure 10B), the ternary mixture exhibits a layered, mesh-like structure. At higher magnification, polycrystalline naringenin appears scattered across a sheet-like matrix with distinctive round, plate-like motifs. Similar motifs were observed in protein controls at pH 7, suggesting they may result from the combined effects of pH and heat treatment. At this pH, NaCas formed a film-like structure, and the circular plate-like features on the surface likely originated from large ice crystals formed during freeze-drying. Similar observations were reported by Kamigaki [57] in high-pressure freezing with cryo-scanning electron microscopy images for raw milk.

**Figure 12:**
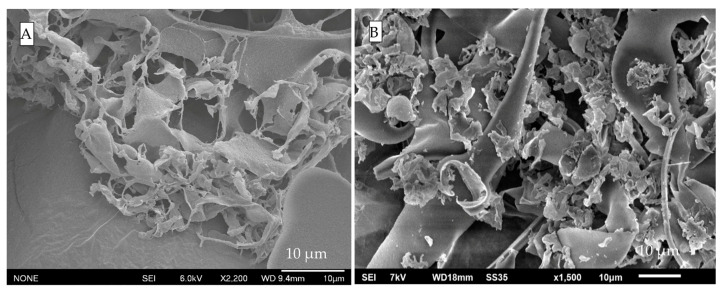
(**A**): Ternary system (basil seed gum water-soluble extract, sodium caseinate, and naringenin) at pH 4, showing a mesh-like microstructure (×2200 magnification). (**B**): Protein binary control at pH 5 displaying aggregated structures (×1500 magnification).

However, it was absent in pH 4 ternary mixtures (Figure 12), even though the layered structure remains, which could prove the hypothesis that polysaccharides cover the proteins during the treatment. When compared to control samples, ternary systems at pH 4 showed minimal denaturation, probably due to the interactions between polysaccharides and also because of the steric hindrance delivered by polysaccharides. Absence of denatured proteins, less ordered structure of nanocrystals, reduced particle size, the stabilization effect provided by the polymer combination, and increased wettability owing to polysaccharides collectively ensure the solubility of the ternary system.

Furthermore, the gel structure in the ternary structure (pH 7) appears to have resulted primarily from polysaccharides, owing to their semi-rigid and organized backbone architectures, as evidenced by SEM imaging (Figure 3E) and the formation of hydrogen bonds under treatment conditions at pH 7. Upon further acidification (pH 4), the structure becomes tighter and more organized, with no observable sedimentation—a phenomenon attributed to the stabilizing effect of polysaccharides. It has been suggested that reduced flexibility and lower entropy, as provided by ordered structures such as β-sheet poly(amino acid)s, contribute to enhanced encapsulation efficiency for bovine serum albumin [43]—a finding that may also help explain the observations in this study.

### 3.6. Flow Curves

**Figure 13:**
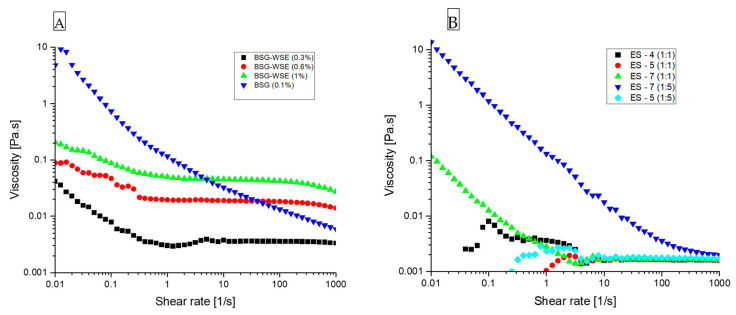
(**A**): Flow curves of different concentration of basil seed gum water-soluble extract (*w*/*v* %) in comparison with basil seeds gum (0.1% g/mL), (**B**): flow curves of different ternary systems-basil seeds gum-sodium caseinate-naringenin at different pH treatments and biopolymer ratios (ES-pH (biopolymer ratio)).

Figure 13A shows the flow curves of BSG-WSE extract at three different concentrations, as well as BSG at 0.1% *w*/*v*. Even at a very low concentration like 0.1%, BSG exhibits pseudoplastic behavior throughout the applied shear force (*n*= 0.537 ± 0.016, K= 0.819 ± 0.025), whereas BSG-WSE shows pseudoplastic behavior only at very low shear rates at 20 °C (all samples were allocated resting time to reach the equilibrium before starting the measurements).

For ternary mixtures (Figure 13B), variations in pH and biopolymer ratios produced distinct flow curves, reflecting differences in particle conformation. At pH 7, samples with both ratios (1:1 and 1:5) behaved as gels, exhibiting pseudoplastic behavior. This likely explains the larger particle size observed at pH 7, indicative of gel formation. Preliminary studies on BSG-WSE during heating–holding–cooling cycles showed a marked increase in storage modulus over time, suggesting enhanced hydrogen bonding and hydrophobic interactions that promote gel development. Similar behavior was noted for basil seed gum, indicating that the heat treatment applied in this study likely contributed to gel particle formation.

Samples at pH 4 and 5 (1:1 ratio) demonstrated Newtonian behavior with comparatively lower viscosities, and at very low shear rates, the curves were not smooth. This also suggests the aggregation behavior (colloidal particles) observed at these particular pH values. On the other hand, lower and more consistent viscosity implies improved reproducibility. In terms of concentration values, BSG-WSE concentration was as low as 0.075% *w*/*v*, and protein concentrations were 0.075% *w*/*v* (1:1 ratio) and 0.0375% *w*/*v* (1:5 ratio). Similar flow behavior has been observed by Sarabi-Aghdam, Hosseini-Parvar, Motamedzadegan and Razi [15] for the BSG-WPI combination.

**Figure 14:**
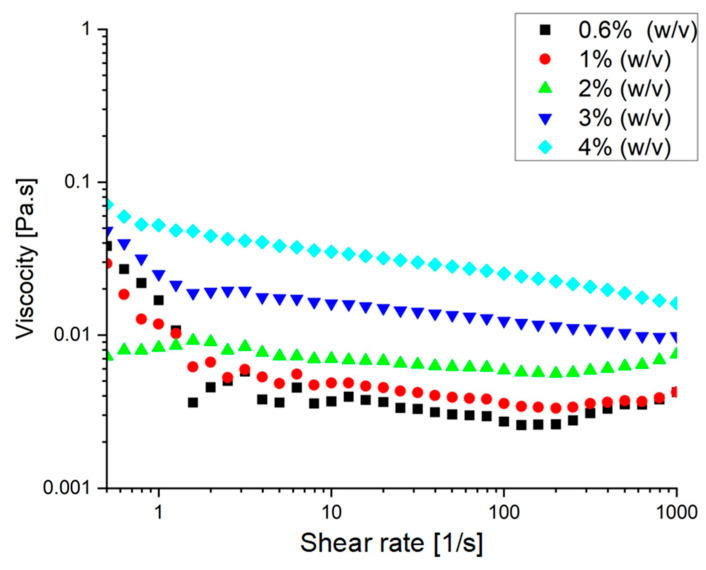
Flow curves of the ternary system (basil seeds gum water-soluble extract–sodium caseinate–naringenin) at pH 4 and at different concentrations.

The ternary system at pH 4 was further examined under varying shear rates (Figure 14), and the formulations in the range of 3–4% (*w*/*v*) exhibited pseudoplastic (shear-thinning) behavior. Pseudoplasticity reflects a high viscosity at rest that decreases with increasing shear. Studies have shown that when incorporated into an emulsion system, such behavior increases the viscosity of the continuous phase, thereby slowing droplet movement and reducing collision frequency [58]. This rheological profile is advantageous for emulsion stabilization, as increased viscosity can delay the thinning and rupture of the liquid film between approaching droplets. Additionally, the colloidal particles formed within the ternary system carry a high negative charge. When adsorbed at the droplet interface, this contributes further stabilization by generating electrostatic repulsion, preventing close approach and aggregation of droplets. Overall, the shear-thinning behavior and high surface charge of these pH 4 ternary complexes suggest strong potential as stabilizing agents in emulsion-based delivery systems.

### 3.7. pH Stability of the System

**Figure 15:**
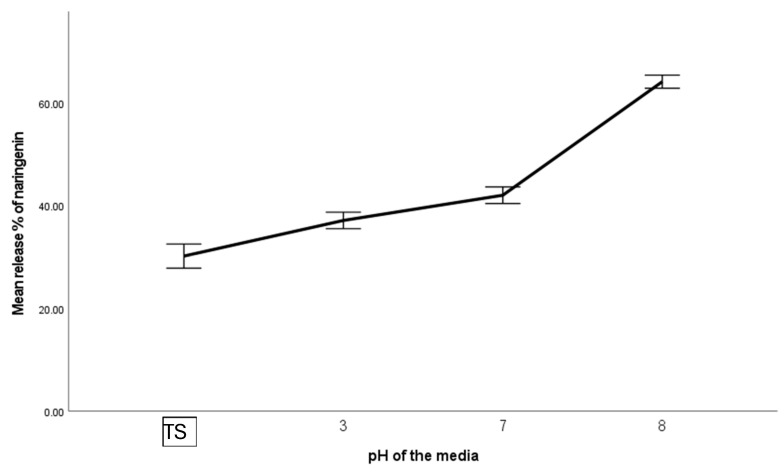
Percentages of free/unencapsulated naringenin at different buffer solutions (3; 37.06% ± 1.39, 7; 41.97% ± 1.39, and 8; 64.08% ± 1.10), compared to the freeze-dried powder. TS: Ternary system of basil seeds gum water-soluble extract, sodium caseinate, and naringenin dissolved in distilled water.

The freeze-dried powder of the pH 4 system was dissolved in different pH media to measure the release of naringenin (the permeates of 10 MWCO filter units were considered as the released amount). As shown in Figure 15, it was observed that a higher amount of naringenin was released at alkaline pH values, suggesting pH-triggered release. It indicates that the ternary system has the potential to protect entrapped naringenin in the stomach, avoiding premature release, since limited release was observed without it in the acidic buffer in the absence of digestive enzymes. The protein in the system aggregates more under the stomach pH level, avoiding the disintegration of the system. Particle size measurements and zeta potential values also aligned with the observation by showing higher particle size and lower negative zeta potential, probably due to increased aggregation at lower pH. The rearrangement that might occur in the ternary system, under acidic pH, could probably release some extra amount of naringenin, indicated by the higher release percentage at pH 3 compared to the ternary system (~4 pH).

On the other hand, since it releases a high percentage of naringenin at alkaline pH due to the disruption of the balance in electrostatic and hydrogen bonding of the ternary system, it indicates that intestinal pH level may trigger the release of entrapped naringenin. Additional factors, such as swelling of the polysaccharide network and ionization of naringenin, may also contribute to the observed release behavior. A previous study [53] indicated that the absorption of naringenin is higher in the small intestine, followed by the colon and stomach, indicating that the proposed system is effective since it can preserve naringenin in the stomach while releasing more in the intestine, facilitating a high dosage. It is well known that naringenin is further metabolized by microbiota present in the gut [9]. This proposed system, combined with BSG-WSE polysaccharides, may serve as a source of prebiotics to facilitate microbial metabolism of the attached naringenin; however, further studies are needed to elucidate the full potential.

## 4. Conclusions

Overall, the ternary system formulated at pH 4 achieved an encapsulation efficiency of 71.55 ± 0.77% and the highest loading capacity (around 11.2%) at a 1:1 biopolymer ratio in the presence of HCl, forming a stable colloidal dispersion through controlled biopolymer complexation. The resulting particles exhibited a mean size of approximately 386 nm with a polydispersity index indicative of a submicron population, together with a ζ-potential exceeding −30 mV, confirming high colloidal stability. System performance was markedly influenced by formulation and processing parameters, including pH, temperature, acidification method, protein content, protein type, and the nature of the hydrophobic flavonoid. Across all tested conditions, basil seed gum water-soluble extract (BSG-WSE) consistently enhanced stability, underscoring its robustness as a functional stabilizing agent.

At pH 7, the ternary formulations formed gel-like structures and exhibited consistently lower encapsulation efficiencies across all biopolymer ratios. Notably, the ternary system significantly improved the aqueous solubility of naringenin at all investigated pH values. The biopolymer ratio (BSG-WSE:NaCas) emerged as a critical determinant of encapsulation performance, with higher protein content yielding greater encapsulation efficiencies, reflecting the strong affinity of naringenin for protein-based binding domains. Although the highest encapsulation efficiency (~79.5%) was achieved at pH 5 with a 1:5 ratio, resulting in soluble complexes, this condition was associated with a comparatively low loading capacity (~4.3%), highlighting an important trade-off between encapsulation efficiency and payload. In vitro release studies conducted in a simulated gastrointestinal buffer system in the absence of digestive enzymes demonstrated the pH-responsive nature of the ternary complexes. Complementary FTIR analysis and pH-dependent release behavior suggest that electrostatic interactions, hydrophobic associations, and hydrogen bonding collectively govern complex formation and stabilization. This strategy represents a viable alternative to conventional pH-driven solubilization approaches, and may help address their inherent limitations. The observed pH responsiveness, combined with submicron-scale assembly using food-grade biopolymers, indicates strong potential for enhancing the bioaccessibility of hydrophobic flavonoids such as naringenin. Furthermore, the favorable rheological properties of the system suggest suitability for incorporation into emulsions or semi-solid food matrices (e.g., yogurt) without compromising textural or functional stability.

Despite these promising findings, this study did not evaluate the effects of ionic strength, mixing sequence, or biopolymer conformational changes during complexation, which represent important limitations. Future work in our laboratory will focus on elucidating the impact of digestive enzymes on release behavior, as well as assessing the performance of this delivery system in model food applications to further establish its translational relevance.

## Figures and Tables

**Table 1:** Proximate composition of basil seeds gum water-soluble extract.

Component	Amount Present
Ash	19.1% ± 0.3
Crude protein	11.45% ± 1.62
Fat	1.55% ± 0.21
Calcium	2.5 mg/g
Glucuronic acid	41.17% ± 14.28

**Table 2:** Size, zeta potential, and PDI measurements of different ternary systems. NA: not analysed. Different superscript letters indicate significant differences at *p* ≤ 0.05 (*n* = 3).

pH	Ratio	Size (nm)	PDI	Zeta Potential (mV)	Encapsulation Efficiency (%)
7	1-1	3410 ± 6.42 ^a^	NA	−32.98 ± 0.28 ^a^	34.76 ± 1.00 ^a^
	1-3	1150 ± 0.01 ^b^	NA	−19.44 ± 0.94 ^b^	35.88 ± 1.09 ^a^
	1-5	886.67 ± 5.77 ^b^	NA	−17.95 ± 0.93 ^b^	40.48 ± 1.64 ^b^
5	1-1	172.40 ± 2.15 ^c^	0.26 ± 0.21	−24.75 ± 1.88 ^c^	62.11 ± 1.41 ^e^
	1-3	196.47 ± 2.31 ^d^	0.29 ± 0.00	−28.46 ± 0.69 ^d^	66.75 ± 1.39 ^f^
	1-5	168.3 ± 1.38 ^e^	0.29 ± 0.02	−25.58 ± 0.32 ^c^	78.38 ± 2.25 ^g^
4	1-1	386.16 ± 3.38 ^f^	0.53 ± 0.00	−29.82 ± 0.80 ^d^	71.55 ± 0.77 ^h^
	1-1 (GDL)	195.52 ± 1.26 ^d^	0.25 ± 0.01	−31.49 ± 0.89 ^d^	72.50 ± 0.85 ^h^
	Wall material	318.16 ± 1.25 ^g^	0.49 ± 0.01	−36.6 ± 5.39 ^f^	-

**Table 3:** Proposed functional groups present in the BSG-WSE (basil seeds gum water-soluble extract) based on the FTIR spectra obtained [45,46,47,48].

Peaks	Possible Interpretation
Broad peak around 3370	OH-stretching
2800–3000	CH-stretching of methyl or methylene groups (intermolecular H bond), acetylated or methyl-esterified CH_3_ groups
1600–1650	Free Carboxylic groups or water bending
~1410	CH_2_ crossover or symmetric stretching of esterified CH_3_
~1250	The stretch vibration of carbonyl CO
~1150	Ether bond (C-O-C) in the polysaccharide chain or CO stretching of primary alcohols
~890	β-glycosidic linkages

## Data Availability

The original contributions presented in the study are included in the article, further inquiries can be directed to the corresponding author.
